# Two-step movement of tsunami boulders unveiled by modified viscous remanent magnetization and radiocarbon dating

**DOI:** 10.1038/s41598-022-17048-8

**Published:** 2022-07-29

**Authors:** Tetsuro Sato, Masahiko Sato, Masaki Yamada, Hirotake Saito, Kenji Satake, Norihiro Nakamura, Kazuhisa Goto, Yosuke Miyairi, Yusuke Yokoyama

**Affiliations:** 1 Department of Earth Science, School of Education, Waseda University, 1-104 Totsukamachi, Shinjuku-ku, Tokyo, 169-8050 Japan; 2 Earthquake Research Institute, The University of Tokyo, 1-1-1 Yayoi, Bunkyo-ku, Tokyo, 113-0032 Japan; 3 Department of Earth and Planetary Science, Graduate School of Science, The University of Tokyo, 7-3-1 Hongo, Bunkyo-ku, Tokyo, 113-0033 Japan; 4 Department of Geology, Faculty of Science, Shinshu University, 3-1-1 Asahi, Matsumoto, Nagano 390-8621 Japan; 5 Institute for Excellence in Higher Education, Tohoku University, 41 Kawauchi, Aoba-ku, Sendai, Miyagi 980-8576 Japan; 6 Atmosphere and Ocean Research Institute, The University of Tokyo, 5-1-5 Kashiwanoha, Kashiwa, Chiba 277-8564 Japan; 7 Graduate Program on Environmental Sciences, Graduate School of Arts and Sciences, The University of Tokyo, 3-8-1 Komaba, Meguro-ku, Tokyo, 153-8902 Japan; 8 Japan Agency for Marine-Earth Science and Technology, Biogeochemistry Research Center, 2-15, Yokosuka, Kanagawa 237-0061 Japan; 9 Research School of Physics, The Australian National University, Canberra, ACT 02000 Australia

**Keywords:** Palaeomagnetism, Geology, Natural hazards, Geomorphology, Geophysics

## Abstract

Massive boulders in landslide and tsunami deposits are prominent geomorphic features in various landscapes. Tracking their movement history is important for reconstructing past geologic dynamics; however, the reworking movements of massive boulders remain unresolved. The boulder field on the Ishigaki Island was formed by repeated tsunamis. Although the individual movement histories of boulders contribute to retrodict the history of different magnitude tsunamis, their radiocarbon ages only correspond to the tsunamis that detached boulders from the reef. Viscous remanent magnetization dating methods have been applied in reworking movements. These methods reveal signals associated with remanent magnetization that gradually grew since the reworking event, which helps to determine the passage of time. The methods were verified by comparison to the radiocarbon ages of un-reworked boulders detached by the recent Meiwa tsunami, while the estimated ages of such two boulders based on the classical relaxation theory contradicted the radiocarbon ages. Here, we show that a method based on the stretched exponential function addressed this contradiction. The reworking movement was estimated using an additional boulder, whose, using our method, radiocarbon age indicated that an older tsunami moved it, whereas the remanent magnetization age unveiled a reworking of the boulder attributed to the Meiwa tsunami.

## Introduction

Extreme marine flooding of coastlines due to storms and tsunamis transports coarse sediments onshore. Sedimentological evidence has been studied at various locations worldwide to estimate the magnitude and occurrences of past extreme flooding events^1–3^. The age of past marine flooding hazards is important for future disaster prediction and mitigation. Although several dating techniques such as optically stimulated luminescence, tephrochronology, and radiocarbon (^14^C) are widely applied to fine sediment records^4^, the dating of wave-emplaced boulders remains a challenge^5,6^. For example, ^14^C, uranium/thorium, and electron spin resonance dating of boulders rely on the availability of datable organisms and the assumption that their death is synchronous with the boulder transport and not related to changes in the relative sea level independent of the marine flooding. Another method, cosmogenic radionuclide dating, relies on freshly exposed surfaces. Complex boulder transport histories with stepwise movement during several successive events cannot be unlocked using any of these approaches. Paleomagnetic information has been used as a marker of such rock displacement[e.g.,^7,8^]. Viscous remanent magnetization (VRM) dating is not only complimentary to the above-mentioned dating techniques, but also overcomes the above-mentioned challenges and can be applied to boulders that have not datable^9,10^.

Rocks contain ferromagnetic minerals that record the geomagnetic field at the time of their formation. After the rock is transported by a marine flooding hazard and the new location is fixed within the geomagnetic field, a subset of constituent magnetic particles is expected to acquire a VRM that aligns with the prevailing geomagnetic field. Pullaiah et al.^11^ determined the temporal stability of the remanent magnetization of ultrafine and uniformly magnetized particles called single-domain (SD) particles^12,13^. The Pullaiah nomogram links the unblocking temperatures determined in the laboratory to theoretical room-temperature relaxation times. The single time–temperature curve corresponds to the SD grain size. These curves have been utilized to date archaeological and geological events during which rocks were rotated relative to the geomagnetic field^9,10,14^. However, the remanent magnetization age is generally older than that obtained from other dating techniques and historical records due to high unblocking temperatures^9,14^. The remanent magnetization of rocks is not only attributed to SD particles but also to non-SD particles (i.e., vortex-state and multi-domain, MD, particles); moreover, the non-SD effects are considered to be the cause of the older ages. The temporal stability curves of uniform vortex-state domain and MD particles are much shallower and yield higher unblocking temperatures than the Pullaiah nomogram^15,16^. Hence Sato et al.^17^ considered the behavior of an assembly of magnetic particles with various domain states and reported that the relaxation of geologic samples is often described in terms of the stretched exponential or Kohlrausch–Williams–Watts function^18,19^. The function can be expressed as a superposition of simple exponential relaxation terms. The rugged energy landscape has been considered, which is unlikely to be shown in the SD particles[e.g.,^20,21^]. The authors suggest that a modified temporal stability of remanent magnetization reconciles the discrepancies between the ages obtained using VRM dating and those obtaining using other dating techniques.

Large coral limestone boulders transported by tsunamis are dispersed across the shore of Ishigaki Island, Japan [e.g.,^1^]. Based on numerical modeling of the movement of a single very large boulder (heavier than 500 metric tons), at least one large tsunami equivalent to or even larger than the historical large tsunami (AD 1771 Meiwa tsunami) occurred before AD 1771^22^. The boulder might have been moved by two tsunami events. Araoka et al.^23^ reported the depositional ages of single *Porites* colony boulders by ^14^C dating, and the recurrence interval of tsunamis was 150–400 years within the past 2250 years. The ^14^C ages indicated that the boulders may have been reworked by successive tsunami events, although the ^14^C age corresponds only to the event that detached the boulder from the reef^23^. Therefore, to determine both the recurrence and scale of past tsunamis, reconstructing the reworked history of the boulders is important. Sato et al.^9,17,24^ applied VRM dating to tsunami boulders on Ishigaki Island and experimentally determined VRM unblocking temperatures, which appeared to be approximately 10 °C higher than those corresponding with the Pullaiah nomogram. Although modified temporal stability curves have the potential to yield reworking ages for such anomalous unblocking temperatures that are consistent with the associated ^14^C ages^24^, it is necessary to measure the time-dependence of the VRM of boulder samples. Furthermore, in previous studies^9,17,24^, it was not straightforward to obtain the comparable ^14^C ages since studied tsunami boulders were composed of accumulations of small coral colonies and each fragment yielded different ^14^C ages^25^. Therefore, in this study, a modified VRM dating protocol was applied to single massive coral boulders on the east coast of Ishigaki Island. We performed a series of time-dependent VRM experiments to describe the relaxation behavior of the boulders. The ^14^C ages of the same specimens that were used for VRM dating were directly compared to the remanent magnetization ages to verify the method and estimate the reworking.

## Methods

### Theory of the modified nomogram and dating protocol

The traditional Pullaiah nomogram is based on Néel’s relaxation theory for SD particles^11–13^. The curves cannot be applied to samples that include non-SD effects^26–28^. Although Walton^26^ developed a theory regarding VRM acquisition for assemblies of SD particles with different volumes and coercivity, it has been pointed out that the physical meaning of the equations is the replacement of a previous VRM acquired at a temperature over a certain time by a new VRM with equal intensity obtained at a higher temperature over a shorter time period^10,29^. On the other hand, Berndt and Chang^15^ advanced previous MD theories by considering the effect of repeated domain wall jumps over many pinning sites, which implies that the VRM stability of MD remanent magnetization. Nagy et al.^16^ numerically derived Pullaiah curves for vortex-state structures. Although currently existing approaches have their unique advantages, tsunami boulders are expected to contain magnetic grains with variable sizes (e.g., SD, vortex state, and MD grains)^24^.

In such situations, the basic idea of stretched exponential relaxation is to assume that a sample comprises an ensemble of domains with various sizes^20^. A simple explanation of the stretched exponential function is that it is the sum of single exponential functions. It should be considered that an aggregate of magnetic particles has various thermal activation energies depending on its shape and size. The continuous-time random walk model can be used to explain the complex energy barriers of such an aggregate. In contrast to the general random walk model, the magnetic particle must wait for a certain time at the local energy minima before each jump can occur (Supplementary Text)^30^. There are waiting times before the jumps of particles in local energy minima can occur in the rugged energy landscape^21^, which is similar to the energy landscape of MD particles^15^. However, in the coarse-grained landscape of energies, the organized sets of energy barriers create deep valleys and the saddle point corresponds to overturning magnetic spins of large clusters (Fig. S1). Thus, rugged energy landscapes might be responsible for slow relaxation. Many particle rearrangements of magnetic moments are necessary for the system to relax (Supplementary Text).

The decay of the remanent magnetization follows a stretched exponential function:

1 $$M\left( t \right) = M_{0} \exp \left\{ { - \left( {\frac{t}{{\tau_{kww} }}} \right)^{\beta } } \right\} {\text{with,}} \, 0 < \beta \le 1,$$

where $M(t)$ is the magnetization at time $t$, ${M}_{0}$ is the original remanent magnetization intensity, ${\tau }_{kww}$ is the characteristic relaxation time, and $\beta$ is the stretching exponent, where $\beta$ = 1 for identical SD particles and 0 $<\beta <$ 1 for aggregates with a broad relaxation time distribution[e.g.,^31^]. The exponent $\beta$ is used as a dispersion factor from an aggregate of identical SD grains^17^, and the curvature (shape) of the relaxation and stability curves for VRM dating are quantified. Following Sato et al.^17^, the relaxation time, which depends on the temperature, is scaled by the stretching exponent $\beta$ and can be calculated as follows:

2 $$\tau^{*} \equiv \tau_{kww}^{\beta } = \left\{ {\frac{1}{C}{\text{exp}}\left( {\frac{{vh_{c} M_{s} \left( T \right)}}{2kT}} \right)} \right\}^{\beta } ,$$

where $v{h}_{c}{M}_{s}\left(T\right)/2$ is the height of the energy barrier $E$, ${M}_{s}\left(T\right)$ is the saturation magnetization, ${h}_{c}$ is the coercivity, $v$ is the effective particle volume, $T$ is the temperature, $k$ is the Boltzmann constant, and $C$ is a frequency factor, which generally ranges between 10^9^ and 10^10^ Hz^9,32^. Because $\beta$ is the dispersion factor of an ideal aggregate of SD particles, the scaled ${\tau }^{*}={\tau }_{kww}^{\beta }$ replaces the longer relaxation time ${\tau }_{kww}$ to yield a shorter relaxation time. If an aggregate of magnetic particles with certain $v$ and ${h}_{c}$ values has a relaxation time ${t}_{A}^{*}$ at temperature ${T}_{A}$, then its relaxation time ${t}_{D}^{*}$ at ${T}_{D}$ is:

3 $$\frac{{T_{A} {\text{ln}}\left\{ {C\left( {t_{A}^{*} } \right)^{{\frac{1}{\beta }}} } \right\}}}{{\left( {T_{C} - T_{A} } \right)^{2y} }} = \frac{{T_{D} {\text{ln}}\left\{ {C\left( {t_{D}^{*} } \right)^{{\frac{1}{\beta }}} } \right\}}}{{\left( {T_{C} - T_{D} } \right)^{2y} }},$$

where ${T}_{C}$ is the Curie point and $y$ is the temperature-dependent exponent for ${M}_{s}\left(T\right)$ (e.g., 0.43 for magnetite)^33^. Once $\beta$ is determined by the measurement of the magnetic time dependence, the unknown ${t}_{A}^{*}$ is estimated to be the true time at ambient temperature ${T}_{A}$ when the demagnetized temperature of ${T}_{D}$ in the absence of a magnetic field is known through thermal demagnetization experiments with a duration time of ${t}_{D}^{*}$^9,24^.

### Samples and ^14^C dating

**Figure 1:**
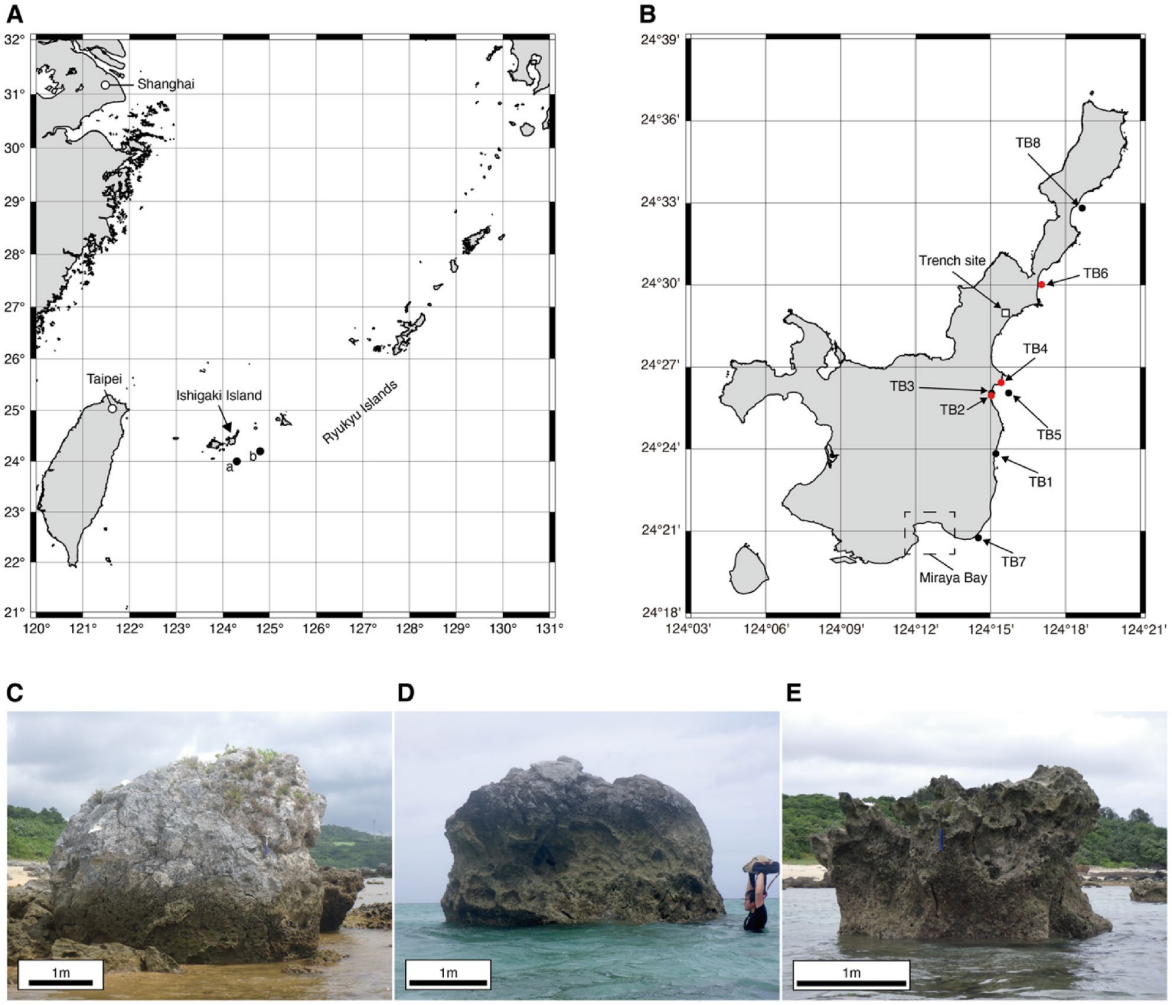
Map of sampling location and sample photos of tsunami boulders. (**A**) Location of Ishigaki Island. Locations a (N24°12, E124°48) and b (N24°, E124°18) denote the epicenters of the earthquake generating the Meiwa tsunami estimated by Imamura^54^ and Hatori^55^, respectively. (**B**) Map of Ishigaki Island and the sampling locations (longitude: E124°14.51–E124°18.67 and latitude: N24°20.75–N24°32.82). Red circles represent utilized thermal demagnetization data and black circles indicate boulders that were rejected because the demagnetization data were noisy. The white square is the trench site reported by previous studies^49–51^. The dashed square (Miyara Bay) is the main field of previous VRM dating studies^9,17,24^. The two maps (**A** and **B**) were generated using the Generic Mapping Tools 6.2.0 (https://www.generic-mapping-tools.org/)^52^. (**C**) TB2. (**D**) TB4. (**E**) TB6.

The youngest, non-eroded parts of single-colony *Porites* boulders were identified in the field by the presence of surface bumpiness^34^. We collected block samples from eight relatively large tsunami boulders (TB1–8) on the eastern shoreline of Ishigaki Island in May and June 2021 (Fig. 1A and B). Field observations confirmed that those boulders had been moved, and their coral growth textures are not in the normal upward direction (e.g., Fig. 1C–E). One-inch core samples were drilled from the block samples for thermal demagnetization and small chip samples were cut from the drilled samples for magnetic measurements.

^14^C dating samples were obtained from the same blocks as those used for VRM dating experiments and were crushed into sub-centimeter-sized pieces. Geochemical treatments and Accelerator Mass Spectrometry ^14^C dating were performed at the Atmosphere and Ocean Research Institute at the University of Tokyo, Japan^35,36^. The ^14^C ages were calibrated using a calendar timescale and the software OxCal 4.2.4^37^ based on comparisons with Marine 20 data after applying a local correction of ΔR =   –36 ± 78^38^.

### Thermal demagnetization and relaxation measurements

Thermal demagnetization (ThD) using a TDS-1 thermal demagnetizer (Natsuhara Giken Co. Ltd., Osaka, Japan) was performed to characterize the VRM and determine ${T}_{D}$ in Eq. (3). The samples were progressively demagnetized by heating at 10 °C temperature steps from 80 to 100 °C, at 5 °C temperature steps from 100 to 200 °C, and at larger steps (i.e., 210, 220, 240, 260, 300, and 350 °C) up to 400 or 500 °C. The temperature was held for 10 min and the samples were cooled to room temperature before the measurement. The superconducting interference devise magnetometer (Model 755, 2G Enterprises, USA) at the University of Tokyo was used for the remanent magnetization measurements during the stepwise ThD treatments.

We measured the time-dependence (relaxation) of the VRM to estimate the $\beta$ value in Eq. (1). The obtained values provide suitable temporal stability curves in Eq. (3). The remanent magnetization of coral limestones was too weak to measure in the 50 μT and 10 mT magnetic field; therefore, a 30 mT field was applied for one hour to achieve VRM. The VRM of boulders with time was measured at 127 °C (400 K) with the Magnetic Properties Measurement System (MPMS3; Quantum Design, USA) at the Cryogenic Research Center at the University of Tokyo. The remanent magnetization intensity was normalized based on the original intensity and the best-fit $\beta$ values were calculated. The measurement conditions of the time-dependence of the VRM (1 h and 127 °C) and the heating time of ThD correspond to the similar unblocking range of tsunami boulders based on the Pullaiah nomogram^24^.

### Magnetic minerals

Low-temperature measurements of remanent magnetization of the two chip samples (TB4 and 6) were conducted using the MPMS3 at the Cryogenic Research Center at the University of Tokyo. The isothermal remanent magnetization (IRM) was imparted at 2.5 T and 10 K after zero-field cooling from 300 K. The remanent magnetization was measured during warming in the zero field (ZFC measurement). Subsequently, the samples were cooled to 10 K in a 2.5 T field and the remanent magnetization was measured during warming in the zero field (FC measurement). The FC and ZFC remanent magnetization curves for the two samples show continuous remanent magnetization decay during the warming to room temperature; low-temperature magnetic transitions at 35 K (pyrrhotite), < 120 K (titanomagnetite), and 260 K (hematite) cannot be observed in the remanent magnetization curves (Fig. S2A and B). Magnetic mineral assemblages with concentrations of superparamagnetic particles undergo magnetic unblocking during warming, whereas superparamagnetic particles do not contribute to the remanent magnetization at room temperature. We also measured the cooling and warming of an IRM imparted in a field of 2.5 T at a temperature of 300 K (RT-IRM). Irreversible cooling and warming curves of the RT-IRM were obtained for the samples. The cooling and warming curves of the RT-IRM exhibit humps and remanent magnetization loss (irreversibility between cooling and warming), which is a typical feature of partially oxidized magnetite particles (Fig. S2C and D)^39^.

## Results and Discussion

### ^14^C ages

**Figure 2:**
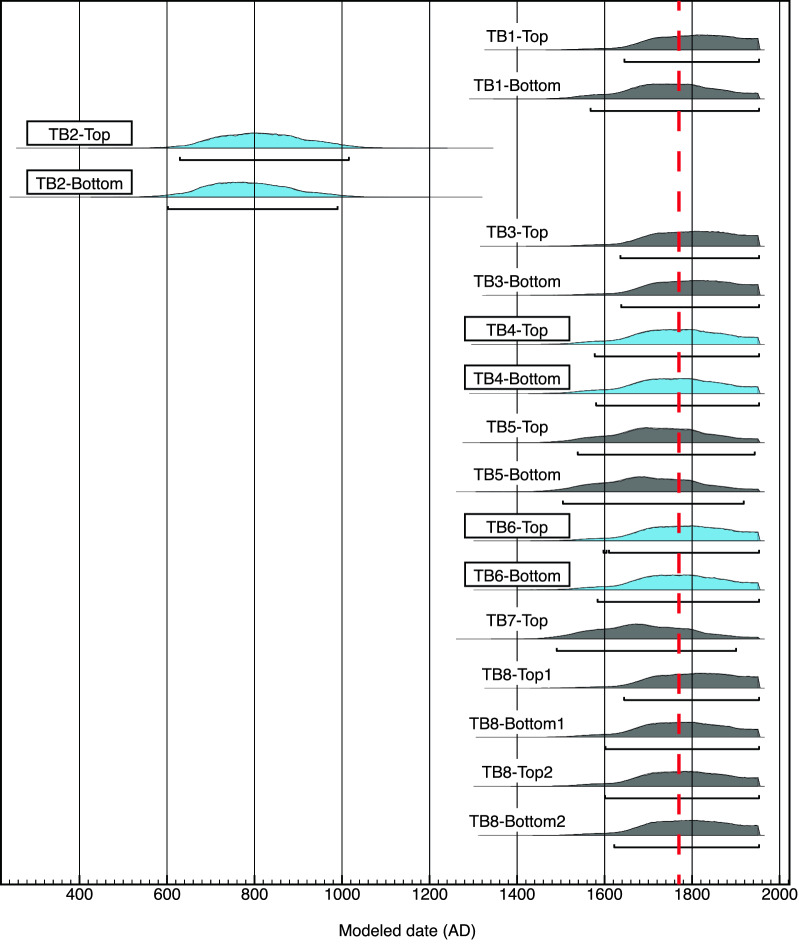
Calibrated ^14^C ages of tsunami boulders. The horizontal black bars are the two-sigma age ranges of the samples. The red line shows the age of the Meiwa tsunami, while the blue color represents utilized thermal demagnetization data samples.

We measured 17 samples from eight tsunami boulders (Fig. 1B). For six of eight boulders, two pieces were taken from the coral surface block and the ages obtained from parts near the coral surface were accepted as the death ages of the coral (Fig. 2, Table 1 and S1). For the other two boulders, one piece from boulder TB7 and four pieces from boulder TB8 were measured. The median ages of the two-sigma age range for six of the eight tsunami boulders are consistent with the Meiwa tsunami within ± 30 yr. The ages of the other boulders are: AD 629–1015 for TB2 and AD 1490–1900 for TB7 (Table 1 and S1). Although the age range of TB7 corresponds to the Meiwa tsunami, the range also covers the AD 1625 event^23^.

**Table 1:** Summary of ^14^C age and VRM analysis of tsunami boulders.

Sample ID	^14^C age (2σ)	Median ^14^C age	Contribution of PCA scores (%)			VRM direction		Unblocking temperature (°C)	$\beta$ value(s)	Error(s) ( ±)
			PC1	PC2	PC3	Declination (°)	Inclination (°)			
TB2-1	AD 629–1015	AD 822	94.2	4.7	1.1	5.7	57.8	180	0.41	0.024
TB2-2	AD 629–1015	AD 822	86.0	9.0	5.0	73.9	68.6	165	0.55	0.045
TB4-1	AD 1577–1953	AD 1765	97.9	1.6	0.4	9.4	30.1	180	0.64 and 0.75	0.0028 and 0.0037
TB4-2	AD 1577–1953	AD 1765	95.4	4.2	0.3	16.4	21.0	155	0.70*	
TB4-3	AD 1577–1953	AD 1765	96.9	2.6	0.5	-2.2	43.1	145	0.71 and 0.71	0.0041 and 0.0029
TB4-4	AD 1577–1953	AD 1765	90.7	7.3	2.0	10.7	35.3	155	0.68	0.0024
TB6-1	AD 1597–1953	AD 1775	83.7	14.6	1.8	2.5	47.9	170	0.63	0.0056

*Average value of samples in the same block.

### Thermal demagnetization

**Figure 3:**
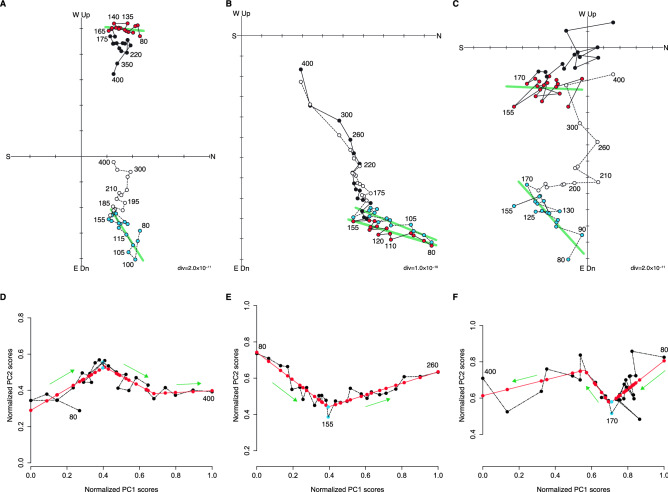
Vector plots and directional analysis of tsunami boulders. (**A**–**C**) Vector plots of tsunami boulders: (**A**) TB2-2, (**B**) TB4-2, and (**C**) TB6-1. Black and red circles represent the horizontal direction and white and blue circles indicate the vertical direction. Red and blue circles represent the VRM component and green lines represent the VRM direction obtained from PCA analysis. (**D**–**F**) Plots of PC1 scores and PC2 scores (black dots with lines) and segmented linear regression results (red dots with lines) for the datasets with scores normalized by the maximum and minimum scores of PC1 and PC2: (**D**) TB2-2, (**E**) TB4-2, and (**F**) TB6-1. The blue dots represent the inflection points.

Stepwise ThD was conducted on 25 core samples from eight tsunami boulders (TB1–8). Seven samples from three boulders were used for directional analysis: two samples from TB2, four samples from TB4, and one sample from TB6 (Figs. 1C–E, 3, S3, and S4). The remaining samples were rejected since they demonstrated noisy data and/or no clear inflection in the demagnetization path. Although several samples from two tsunami boulders (TB4 and 6) showed three principal magnetic components (Figs. 3C and S3), these boulders, which were emplaced by the Meiwa tsunami, should have a VRM component (Fig. 2). Thus, the lower temperature components were accepted as VRM; the high temperature components of these boulders were not assigned as VRM, as they supposedly belong to the primary or secondary magnetizations of the block.

To identify the inflection point (${T}_{D}$) in the demagnetization path, principal component analysis (PCA) was conducted (Table 1). PCA is a multivariate statistical technique used to concentrate the variability in a dataset into a small number of dimensions. Following Sato et al.^24^, the first principal component (PC1) is the mean direction from the first demagnetized point to the last point. The variation of the second principal component (PC2) representing the orthogonal direction of PC1 was caused by an inflection in the demagnetization path. We fit the segmented linear regression of PC1 scores vs. PC2 scores and considered the highest unblocking temperatures on the line to be the inflection points (Fig. 3D–F and Table 1).

The demagnetization plots obtained for three tsunami boulders (TB2, 4, and 6; Fig. 3A–C) indicate a net sideways boulder movement, which has resulted in a horizontal VRM direction not parallel to the older remanent magnetization component, whereas the vertical components of both remanent magnetizations point downward. The field observations of coral growth patterns of tsunami boulders show sideways upward (south upward: TB2 and west upward: TB4) and normal upward (TB6) directions. These patterns indicate that the boulders have been moved via a sliding motion and support the demagnetization plots. The VRM components obtained from PCA are oriented northward (Table 1 and Fig. S4).

### Verification of the Pullaiah nomogram (${\varvec{\beta}}$ = 1) for Meiwa tsunami boulders

**Figure 4:**
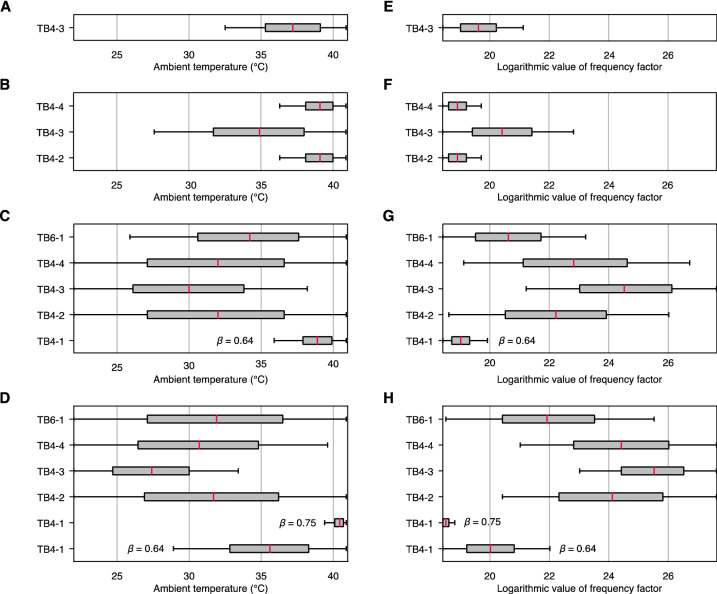
Boxplots of ambient temperature ${T}_{A}$ and frequency factor $C$ variations for Meiwa tsunami boulders (TB4 and 6). (**A**–**D**) ${T}_{A}$ variation and (**E**–**H**) $C$ variation when the VRM ages correspond to the Meiwa tsunami age. The red lines are the median values for each dataset. (**A**) and (**E**) are for magnetite ${T}_{C}$ and (**B**) and (**F**) are for maghemite ${T}_{C}$ based on the Pullaiah nomogram calculation. (**C**) and (**G**) are for magnetite ${T}_{C}$ and (**D**) and (**H**) are for maghemite ${T}_{C}$ based on the modified nomogram calculation.

The remanent magnetization age estimates have a large variance, covering several orders of magnitude, which may be due to the parameter selections in Eq. (3) for the dominant magnetic minerals related to the Curie point ${T}_{C}$, frequency factor $C$, and ambient temperatures ${T}_{A}$, to which samples have been exposed^10,24^. Thus, we examined the parameter selections using TB4 and TB6 because these boulders were transported by the recent Meiwa tsunami, and the VRMs are attributed to the same tsunami. In previous studies, it has been considered that the magnetic mineralogy of the tsunami boulders is dominated by magnetite^9,24,40^. However, the low-temperature magnetic characteristics suggest the existence of the oxidized magnetite particles (Fig. S2), and maghemite is the oxidation or weathering product of magnetite^39^. Although the ${T}_{C}$ changes depending on the oxidation state of particles^41,42^, we calculated two end-member patterns for ${T}_{C}$: magnetite and maghemite (580 °C and 645 °C, respectively)^43^. The saturation magnetization ${M}_{s}\left(T\right)$ of maghemite is not well known due to its thermal instability. However, it should have a similar curve to that of magnetite ($y$ = 0.43)^33,44,45^. Moreover, the surface oxidation process of bare magnetite particles is completed within a short time (several hours) at 50 °C^46^. The tsunami boulders have been emplaced onshore for 100–1000 yr. While the oxidation process of magnetite particles in the coral matrix would need a longer time than that required for bare particles, the time required for the oxidation process is likely much shorter than 100–1000 yr. Thus, the change in the VRM acquisition rate during the time required for gradual oxidation might be negligible. The $C$ is known as the reciprocal of the atomic attempt time, which is an average timescale between two successive random thermal excitations^47^. The value is a material constant that ranges from 10^8^ to 10^12^ Hz but remains poorly constrained^10,47^. For example, Sato et al.^9^ used 10^10^ Hz, whereas Muxworthy et al.^32^ used 10^9^ Hz. Furthermore, calculating an age relies on estimation of the ${T}_{A}$ over the period of VRM acquisition. The lowest ${T}_{A}$ was estimated as the modern mean minimum temperature (22.4 °C) from January 1981 to December 2020 (data from the Japan Meteorological Agency). To assign the highest ${T}_{A}$, we measured the surface temperature of a boulder at the Miyara Bay beach on Ishigaki Island from 08:45 on August 10 to 18:00 on August 11, 2021 (Fig. S5). Solar radiation heated the boulder in a sunny place at 12:00; the highest temperature of the surface was approximately 50 °C at 12:00 on August 11 and the longest heating time > 45 °C was approximately 3 h (i.e., from 13:35 to 16:15 on August 10). By using the highest observed temperature value and heating time (i.e., 50 °C and 3 h, respectively), we calculated the highest ${T}_{A}$ scenario corresponding to the Meiwa tsunami event based on the Pullaiah nomogram; the steepest temporal stability curve was calculated based on the assumption of magnetite ${T}_{C}$ and $C$ = 10^12^ Hz. The highest ${T}_{A}$ was assigned as 41 °C. Subsequently, we determined whether the estimated ages considering the variation of the three parameters (${T}_{C}$, $C$, and ${T}_{A}$) correspond to the Meiwa tsunami with an uncertainty of the estimated ages of ± 50 yr; the combination of $C$ and ${T}_{A}$ was calculated at two different ${T}_{C}$ (Fig. 4). One sample (TB4-3) corresponds to the Meiwa tsunami based on the assumption of magnetite ${T}_{C}$ and three samples (TB4-2, TB4-3, and TB4-4) correspond to the Meiwa tsunami based on the assumption of maghemite ${T}_{C}$. The ranges of the median values of the ${T}_{A}$ and $C$ were 37.2 °C and 3.3 × 10^8^ Hz (TB4-3), respectively, for magnetite ${T}_{C}$ and 34.9–39.1 °C and 1.6–7.4 × 10^8^ Hz, respectively, for maghemite ${T}_{C}$ (Table 2). However, the parameters $C$ and ${T}_{A}$ for two samples (TB4-1 and TB6-1) did not have values corresponding to the Meiwa tsunami (Fig. 4A, B, E, and F).

**Table 2:** Parameters selected for Meiwa tsunami boulders.

Sample name	Nomogram type	Magnetic mineral	Median ambient temperatures (°C)	Median frequency factors (Hz)	Rejection*
TB4-3	Pullaiah	Magnetite	37.2	3.3 × 10^8^	X
TB4-2	Pullaiah	Maghemite	39.1	1.6 × 10^8^	X
TB4-3	Pullaiah	Maghemite	34.9	7.4 × 10^8^	–
TB4-4	Pullaiah	Maghemite	39.1	1.6 × 10^8^	X
TB4-1 ($\beta$ = 0.64)	Modified	Magnetite	38.9	1.8 × 10^8^	X
TB4-2	Modified	Magnetite	32.0	4.5 × 10^9^	–
TB4-3	Modified	Magnetite	30.0	4.5 × 10^10^	–
TB4-4	Modified	Magnetite	32.0	8.1 × 10^9^	–
TB6-1	Modified	Magnetite	34.2	9.0 × 10^8^	–
TB4-1 ($\beta$ = 0.64)	Modified	Maghemite	35.6	5.0 × 10^8^	–
TB4-1 ($\beta$ = 0.75)	Modified	Maghemite	40.5	1.1 × 10^8^	X
TB4-2	Modified	Maghemite	31.7	3.0 × 10^10^	–
TB4-3	Modified	Maghemite	27.4	1.2 × 10^11^	–
TB4-4	Modified	Maghemite	30.7	4.0 × 10^10^	–
TB6-1	Modified	Maghemite	31.9	3.3 × 10^9^	–

* ‘X’ is the rejected data and ‘–’is the accepted data.

Although several samples can be explained by the Pullaiah nomogram, it remains unclear for ambient temperature as to how often intense heating occurs (e.g., peak summer temperatures). Jenkins and Smith^48^ reported that the daytime rock surface temperature varies; the maximum summer temperature is approximately 10 °C higher than the winter temperature. Significant differences in the absolute temperatures and temperature gradients also occur depending on the orientation of the rock surfaces and degree of cloud cover^48^. We therefore suggest that a high ambient temperature of approximately 40 °C is not suitable for VRM dating. The highest acceptable ambient temperature (${T}_{A}$) is assumed to be < 36 °C (i.e., average of 41 and 31 °C in summer and winter, respectively; Table 2). Almost all unblocking temperatures cannot be explained by the parameter settings and must be further investigated.

### Time dependence of VRM and verification of the modified protocol for Meiwa tsunami boulders

**Figure 5:**
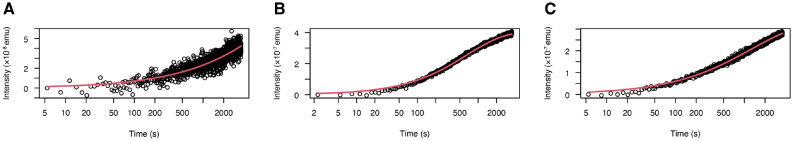
Time dependence of VRM. The circles are data points and the red lines are curve fitting results for the stretched exponential function. (**A**) TB2-2, (**B**) TB4-3, and (**C**) TB6-1.

The time-dependent remanent magnetization curves were measured, and the best-fit curves of the stretched exponential (Eq. 1) were obtained. Because a 30 mT field was applied to the samples, $\beta$ values were used for the acquisition curves to avoid the mixture of VRM and IRM relaxation of the decay curves. The stretched exponential curves agree well with the experimental results (Figs. 5 and S6). The $\beta$ values at 127 °C were used for the age estimation of the modified nomogram (Table 2).

Using the Meiwa tsunami boulders, the parameter selection (${T}_{C}$, $C$, and ${T}_{A}$) for the modified nomogram was analyzed (Fig. 4C,D,G, and H). Based on the modified nomogram (Eq. 3) and the assumption of the ${T}_{C}$ of magnetite, all samples were found to correspond to the Meiwa tsunami, except for TB4-1 with $\beta$ = 0.75 (Table 2). The ranges of the median ${T}_{A}$ and median $C$ values were 30–38.9 °C and 1.8 × 10^8^–4.5 × 10^10^ Hz, respectively. All cases corresponded to the event based on the assumption of maghemite ${T}_{C}$. The ranges of the median ${T}_{A}$ and $C$ values were 27.4–40.5 °C and 1.1 × 10^8^–1.2 × 10^11^, respectively. The modified curves agree better with the Meiwa tsunami event than the Pullaiah nomogram for the ${T}_{C}$ of both magnetite and maghemite, even though the highest acceptable ambient temperature (< 36 °C) is considered (Table 2). The mean value of ${T}_{A}$ calculated from accepted samples (i.e., 32.1 °C for magnetite and 31.5 °C for maghemite) correspond well to the mean of time-integral ${T}_{A}$ (30.7 °C) measured in the field, even though the measurement time was only 33 h (Table 2 and Fig. S5). Furthermore, we considered that the unblocking temperatures documented here are mainly due to maghemite because more samples can be explained by this scenario (i.e., four accepted samples for magnetite and five for maghemite; Table 2).

### Age estimation of an additional boulder and implications for reworking

**Figure 6:**
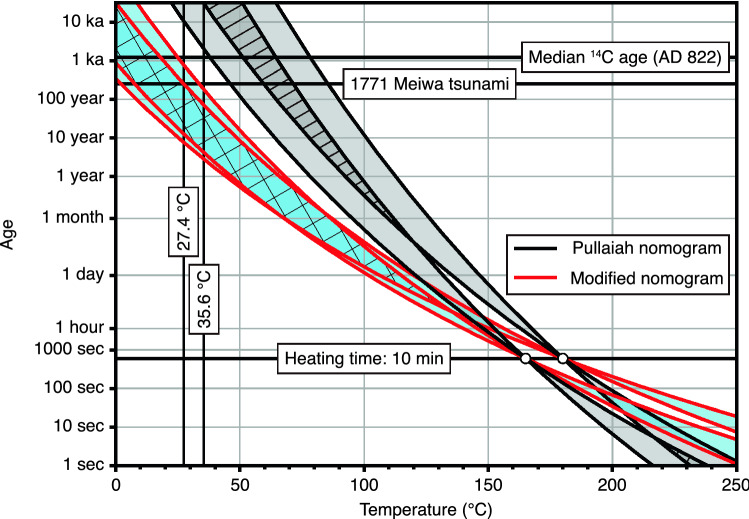
Pullaiah and modified nomograms for the tsunami boulder (TB2). The white circles represent the unblocking temperatures of the samples. The black curves were calculated with the Pullaiah nomogram and red curves represent the modified nomogram. Gray areas indicate the unblocking ranges of the Pullaiah nomogram and the area with hatched lines represents areas overlapping with the gray areas. The blue areas are the unblocking ranges of the modified nomogram and the area with cross-hatched lines represents areas overlapping with the blue areas.

We applied the temporal stability curves of the Pullaiah and modified nomogram to TB2 with a median ^14^C age of AD 822 (Table 1 and Fig. 6). Using the best parameters for the Meiwa tsunami boulders, the two parameters (i.e., ${T}_{C}$ and the median ${T}_{A}$ range) were determined to be 645 °C and 27.4–35.6 °C, respectively (Table 2). The frequency factor $C$ range was assumed to be 10^8^ to 10^12^ Hz. The variations in the estimated age ranges based on the Pullaiah nomogram are: 3.0 × 10^4^–8.8 × 10^8^ yr (TB2-1) and 2.0 × 10^3^–1.8 × 10^7^ yr (TB2-2). The two age ranges based on the Pullaiah nomogram do not coincide and are older than AD 822 (1200 yr ago). The age ranges based on the modified nomogram are: 3–250 yr (TB2-1) and 4–764 yr (TB2-2). The age ranges of the modified nomograms are younger than AD 822, but include the Meiwa tsunami event (AD 1771). Although the ages estimated based on the Pullaiah nomograms could not be explained, reworkings of TB2 might explain the ages of the modified nomograms. Araoka et al.^23^ reported repeated tsunami occurrences and Hisamatsu et al.^22^ suggested that the Meiwa and older tsunami reworked the boulder larger than TB2. Sandy tsunami deposits that extend far inland indicate that several large tsunami events occurred over the last 2000 years^49–51^. The single VRM component of TB2 might be explained by reworkings; if the first boulder movement occurred at AD 822 and successive movements occurred with similar recurrence intervals, TB2 may have a VRM component because the low-coercivity component might have been replaced by the younger event. Therefore, although it is necessary to have a sufficiently large data size, we suggest that the reworking of TB2 occurred during the Meiwa tsunami event.

Numerical calculations incorporating a tsunami boulder size and position are widely used to reconstruct the tsunami size needed to move a boulder. These studies are generally applied to single tsunami event, where large boulders have been moved once and are subsequently unmoved^5^. However, if the mobilization history of a large boulder can be established, it could be used to estimate the different magnitudes of tsunamis where there is a threshold between tsunami size and the size of boulders. VRM dating of boulders may serve as a useful proxy for large-magnitude and low-frequency tsunami events.

In this study, VRM dating was performed on coral tsunami boulders and their reworking ages were estimated based on the unblocking temperatures of VRM. The remanent magnetization ages estimated by the traditional Pullaiah nomogram were too old compared with the ^14^C ages obtained from the same samples. This is due to the basic concept of the relaxation theory of remanent magnetization, which is only valid for strictly ideal SD particles. To address this problem, we applied the modified nomogram, which considers the non-SD effects. Furthermore, based on the analysis, we constrained the parameter selection for VRM dating, especially that of ambient temperatures. The combined use of VRM and ^14^C dating allows for the detection of the reworking events of one boulder. However, several uncertainties remain: (i) our relaxation experimental results describing the $\beta$ values might include non-interacting SD grains with varying sizes due to experimental limitations; in our modified time–temperature curves, a single curve corresponds to an identical SD grain plus non-SD effects; (ii) the frequency factor remains poorly constrained; an experimental approach, such as that of Berndt et al.^47^, may be required to determine the value; (iii) drilling of samples into a boulder might improve results because the mean time-integral ambient temperature value calculated from climate change data can be useful for the age estimation. Despite issues associated with the experimental rock magnetic behavior, our results provide insights into the relaxation behavior, while the temporal stability curves of the magnetization are shown to be improved tools for estimating age of geological events.

## Supplementary Information

Supplementary Information.

## Data Availability

All data are available in the main text or the supplementary materials.
