# Supplementary material for: Two-step movement of tsunami boulders unveiled by modified viscous remanent magnetization and radiocarbon dating
Source: Sci Rep. 2022 Jul 29;12:13011. doi: 10.1038/s41598-022-17048-8 (PMC9338091; doi:10.1038/s41598-022-17048-8)
Supplement: Supplementary file 1 — Supplementary Information. [file 41598_2022_17048_MOESM1_ESM.docx]

Supplementary Materials for:

**Two-step movement of tsunami boulders unveiled by modified viscous remanent magnetization and radiocarbon dating**

Tetsuro Sato^1,2*^, Masahiko Sato^3^, Masaki Yamada^4^, Hirotake Saito^4^, Kenji Satake^2^, Norihiro Nakamura^5^, Kazuhisa Goto^3^, Yosuke Miyairi^6^, Yusuke Yokoyama^3,6,7,8,9^

^1^Department of Earth Science, School of Education, Waseda University, 1-104 Totsukamachi, Shinjuku-ku, Tokyo 169-8050, Japan (tetsuro.sato@aoni.waseda.jp)

^2^Earthquake Research Institute, The University of Tokyo, 1-1-1 Yayoi, Bunkyo-ku, Tokyo 113-0032, Japan

^3^Department of Earth and Planetary Science, Graduate School of Science, The University of Tokyo, 7-3-1 Hongo, Bunkyo-ku, Tokyo 113-0033, Japan

^4^Department of Geology, Faculty of Science, Shinshu University, 3-1-1 Asahi, Matsumoto, Nagano 390-8621, Japan

^5^Institute for Excellence in Higher Education, Tohoku University, 41 Kawauchi, Aoba-ku, Sendai, Miyagi 980-8576, Japan

^6^Atmosphere and Ocean Research Institute, The University of Tokyo, 5-1-5 Kashiwanoha, Kashiwa, Chiba 277-8564, Japan

^7^Graduate Program on Environmental Sciences, Graduate School of Arts and Sciences, The University of Tokyo, 3-8-1 Komaba, Meguro-ku, Tokyo 153-8902, Japan

^8^Biogeochemistry Research Center, Japan Agency for Marine-Earth Science and Technology, 2-15 Yokosuka, Kanagawa 237-0061, Japan

^9^Research School of Physics, The Australian National University, Canberra, ACT 02000, Australia

***Corresponding author:**

Dr. Tetsuro Sato

Department of Earth Science

School of Education, Waseda University

1-104 Totsukamachi, Shinjuku-ku, Tokyo 169-8050, Japan

Phone number: +813-5286-9864

Email: tetsuro.sato@aoni.waseda.jp

This file includes:

Supplementary Text

Supplementary Figures S1 to S6

Supplementary Table S1

Supplementary References

**Supplementary Text: Background and brief review of the stretched exponential function**

Following the theory of Néel^[1,2]^, the acquisition and decay of the VRM of identical SD particles change exponentially with time. The relaxation time, which is a measure of how long it takes for a particle to overcome its energy barrier through thermal activation, follows the Arrhenius–Néel law^[1,2]^. However, the time dependence of the VRM of synthesized magnetic particles and natural rocks is generally nonexponential^[3–5]^. Although an alternative logarithmic time dependence law has been established^[6,7]^, such an approximation is invalid based on experimental results. The law predicts that the magnetization intensity eventually reaches an infinite value. It has been suggested that the presence of nonuniformly magnetized coarse particles in an aggregate of ferromagnetic particles with widely varying sizes and shapes determines the relaxation behavior^[8]^ and the coarser particles with larger domains cause a slower relaxation phenomenon^[9]^. Furthermore, interactions between SD moments are analogous to interactions between domains with domain-wall coercivities^[10]^.

The stretched exponential relaxation function $F\left( t \right)$ takes the form of the sum of exponential decay with respect to the nonuniform distribution of the relaxation time $w\left( \tau\right)$. Montroll and Bendler^[11]^ noted that:

| $F\left( t \right)=\exp\left\{ -\left( \frac{t}{\tau_{kww}} \right)^{\beta} \right\}=\int_{0}^{\infty} w\left( \tau\right)\exp\left( -\frac{t}{\tau} \right)d\tau,$ | (S1) |
| --- | --- |

This attempt has the advantage of retaining the features of independent exponential relaxation processes. Notably, a given empirical relaxation law is compatible with a particular distribution of relaxation times or rates. To derive $w\left( \tau\right)$, Lindsey and Patterson^[12]^ made the following substitutions: $s=t/{\tau_{kww}}$, $\mu={\tau_{kww}}/\tau$, and $\lambda\left( \mu,\beta\right)=\tau_{kww}\mu^{-2}w\left( {\tau_{kww}}/\mu\right)$, where $\lambda\left( \mu,\beta\right)$ is the distribution of the dimensionless relaxation rate $\mu$ and the inverse Laplace transform of $\exp\left( -s^{\beta} \right)$. Thus, it can be rewritten as an integral representation and solved through the gamma ($\Gamma$) function^[13]^:

| $\lambda\left( \mu,\beta\right)=-\frac{1}{\pi}\sum_{k=0}^{\infty} \frac{\left( -1 \right)^{k}}{k!}\sin\pi\beta k\frac{\Gamma\left( \beta k+1 \right)}{\mu^{\beta k+1}}.$ | (S2) |
| --- | --- |

Humbert^[14]^ first showed and Pollard^[15]^ later demonstrated that $\lambda\left( \mu,\beta\right)$ can be written using the closed analytic form in Eq. (S2). Based on the use of $\lambda\left( \mu, \beta\right)=\tau_{kww}\mu^{-2}w\left( {\tau_{kww}}/\mu\right)$, one can obtain a simple relationship for the distribution function $\mu\lambda\left( \mu, \beta\right)=\tau w\left( \tau\right)$. Thus, the distribution of the relaxation times may also follow Eq. (S2). Moreover, Eq. (S2) indicates that the distribution of relaxation times can be obtained by the Levy stable distribution function.

In the section above, various magnetic particles contribute to the relaxation system and their distribution does not follow a particular pattern. In such situations, thermal activation energies also have a distribution because each relaxation time has a thermal activation energy barrier. Random walk processes are powerful tools used to connect the energy landscape and relaxation theory. For general random walks, if a random walker (magnetic particle) strolling in one dimension moves a fixed step of length in either direction when each unit time elapses, the displacement after a large number of steps follows a Gaussian distribution. In contrast, in the continuous-time random walk model, the random walker performs a random walk but must wait for a certain time before each jump; $P\left( x, t \right)$ is the probability density function for locating the random walker at position $x$ and time $t$. In a continuous-time random walk, the probability of a particle arriving at site $x$ at time $t$ is $j_{+}\left( x, t \right)$ and the probability of leaving site $x$ at time $t$ is $j_{-}\left( x, t \right)$. The balance equation at each site is as follows^[16]^:

| $\frac{\partial P\left( x, t \right)}{\partial t}=j_{+}\left( x, t \right)-j_{-}\left( x, t \right).$ | (S3) |
| --- | --- |

$j_{-}\left( x, t \right)$ is given by

| $j_{-}\left( x,t \right)=P\left( x,0 \right)\varphi\left( t \right)+\int_{0}^{t} dt^{'}\varphi\left( t-t^{'} \right)j_{+}\left( x,t^{'} \right)$ $=P\left( x,0 \right)\varphi\left( t \right)+\int_{0}^{t} dt^{'}\varphi\left( t-t^{'} \right)\left\{ \frac{\partial P\left( x,t^{'} \right)}{\partial t}+j_{-}\left( x,t^{'} \right) \right\}.$ | (S4) |
| --- | --- |

The first term of the first line in Eq. (S4) represents a particle that is originally at site $x$ at time zero and leaves at time $t$ and the second term of the first line represents a particle that arrives at $x$ at ${0<t}^{'}<t$ and leaves at time $t$, where $\varphi\left( t \right)$ and $\varphi\left( t-t^{'} \right)$ are the waiting time distribution^[16]^. Equation (S4) is solved by a Laplace transformation^[17]^:

| $\mathcal{L}\left[ j_{-}\left( x,s \right) \right]=P\left( x,0 \right)\mathcal{L}\left[ \varphi\left( s \right) \right]\mathcal{+L}\left[ \varphi\left( s \right) \right]\left\{ s\mathcal{L}\left[ P\left( x,s \right) \right]-P\left( x,0 \right)\mathcal{+L}\left[ j_{-}\left( x,s \right) \right] \right\}=\frac{s\mathcal{L}\left[ \varphi\left( s \right) \right]\mathcal{L}\left[ P\left( x,s \right) \right]}{1-\mathcal{L}\left[ \varphi\left( s \right) \right]}=s\mathcal{L}\left[ \phi\left( s \right) \right]\mathcal{L}\left[ P\left( x,s \right) \right].$ | (S5) |
| --- | --- |

Because the term related to $P\left( x,0 \right)$ vanishes, the inverse Laplace transformation of Eq. (S5) can be expressed as follows:

| $j_{-}\left( x,t \right)=\frac{\partial}{\partial t}\int_{0}^{t} dt^{'}\phi\left( t-t^{'} \right)P\left( x,t^{'} \right).$ | (S6) |
| --- | --- |

The terms $j_{+}\left( x,t \right)$ and $j_{-}\left( x,t \right)$ are satisfied with the balance equation $j_{+}\left( x,t \right)=\sum p\left( x-x^{'} \right)j_{-}\left( x^{'},t \right)$^[18]^, where $p\left( x-x^{'} \right)$ is the jump probability from $x^{'}$ to $x$:

| $\frac{\partial P\left( x,t \right)}{\partial t}=\frac{\partial}{\partial t}\int_{0}^{t} dt^{'}\phi\left( t-t^{'} \right)\left[ \sum p\left( x-x^{'} \right)P\left( x^{'},t^{'} \right)-P\left( x,t^{'} \right) \right]$ $=\frac{\partial}{\partial t}\int_{0}^{t} dt^{'}\phi\left( t-t^{'} \right)\left[ \sum P\left( x^{'},t^{'} \right)\left\{ p\left( x-x^{'} \right)-\frac{P\left( x,t^{'} \right)}{\sum P\left( x^{'},t^{'} \right)} \right\} \right]$ $\approx\frac{\partial}{\partial t}\int_{0}^{t} dt^{'}\phi\left( t-t^{'} \right)\int dx^{'}P\left( x^{'},t^{'} \right)\left\{ p\left( x-x^{'} \right)-\frac{P\left( x,t^{'} \right)}{\int x^{'}P\left( x^{'},t^{'} \right)} \right\}.$ | (S7) |
| --- | --- |

The Taylor expansion of $P\left( x^{'},t^{'} \right)$ then leads to:

| $\frac{\partial P\left( x,t \right)}{\partial t}=\frac{\partial}{\partial t}\int_{0}^{t} dt^{'}\phi\left( t-t^{'} \right)\left\{ \int dx^{'}p\left( x-x^{'} \right)\left( x^{'}-x \right)\frac{\partial}{\partial x}P\left( x,t^{'} \right)+\int dx^{'}p\left( x-x^{'} \right)\frac{1}{2}\left( x^{'}-x \right)^{2}\frac{\partial^{2}}{\partial x^{2}}P\left( x,t^{'} \right) \right\}=\frac{\partial}{\partial t}\int_{0}^{t} dt^{'}\phi\left( t-t^{'} \right)\left\{ A\frac{\partial}{\partial x}P\left( x,t^{'} \right)+B\frac{\partial^{2}}{\partial x^{2}}P\left( x,t^{'} \right) \right\}.$ | (S8) |
| --- | --- |

where $A=\int dx^{'}p\left( x-x^{'} \right)\left( x^{'}-x \right)$ and $B=\int dx^{'}p\left( x-x^{'} \right)\frac{1}{2}\left( x^{'}-x \right)^{2}$. This equation is known as the generalized Fokker–Planck equation, which can be used to describe the rotations of small particles^[19]^, where $\phi\left( t \right)$ is ${t^{\alpha-1}}/{\Gamma\left( \alpha\right)}$ when the waiting time distribution $\varphi\left( t \right)$ is defined as power law [i.e., $\varphi\left( t \right)\propto t^{-1-\alpha}$]^[e.g.,16,20,21]^. The equation is as follows:

| $\frac{\partial P\left( x,t \right)}{\partial t}=\frac{1}{\Gamma\left( \alpha\right)}\frac{\partial}{\partial t}\int_{0}^{t} dt^{'}\left( t-t^{'} \right)^{\alpha-1}\left\{ A\frac{\partial}{\partial x}P\left( x,t^{'} \right)+B\frac{\partial^{2}}{\partial x^{2}}P\left( x,t^{'} \right) \right\}.$ | (S9) |
| --- | --- |

It is considered that the general way to introduce fractional derivatives is based on the fact that the $n$ th derivative is an operation inverse to an $n$ th fold repeated integration^[22]^. Therefore, one can obtain the fractional derivative of the Fokker–Planck equation:

| $\frac{\partial P\left( x,t \right)}{\partial t}={}_{0}{D_{t}^{1-\alpha}}\left\{ A\frac{\partial}{\partial x}P\left( x,t \right)+B\frac{\partial^{2}}{\partial x^{2}}P\left( x,t \right) \right\},$ | (S10) |
| --- | --- |

where the fractional ${}_{a}{D_{y}^{-n}}$ is defined as integration operator. Equation (S10) can be separated through the ansatz $P\left( x,t \right)=X\left( x \right)T\left( t \right)$and $T\left( t \right)$ is solved using the following equation:

| $\frac{\partial T\left( t \right)}{\partial t}=-\varepsilon{}_{0}{D_{t}^{1-\alpha}}T\left( t \right),$ | (S11) |
| --- | --- |

where $\varepsilon$ is the eigenvalue^[23,24]^. If $\varepsilon$ is $\tau^{-\alpha}$ and $\alpha$ is $\beta$, the solution of Eq. (S11) yields the terms of the Mittag–Leffler function $E_{\beta}$ via $T\left( t \right)={T\left( 0 \right)E}_{\beta}$, which describes the relaxation toward the equilibrium of particles^[22]^. $E_{1}\left( {-t}/\tau\right)$ represents a normal exponential relaxation function and $E_{\beta}\left\{ {-\left( t/\tau\right)}^{\beta} \right\}$ exhibits the following limiting behaviors^[25,26]^:

| $E_{\beta}\left\{ {-\left( \frac{t}{\tau} \right)}^{\beta} \right\}=\sum_{q=0}^{\infty} \frac{\left( -1 \right)^{q}}{\Gamma\left( \beta q+1 \right)}\left( \frac{t}{\tau} \right)^{\beta q}\sim\left\{ \begin{matrix} \exp\left\{ \frac{-\left( t/\tau\right)^{\beta}}{\Gamma\left( 1+\beta\right)} \right\},t\ll\tau\\ \frac{1}{\Gamma\left( 1-\beta\right)\left( t/\tau\right)^{\beta}},t\gg\tau\end{matrix} \right..$ | (S12) |
| --- | --- |

The Mittag–Leffler function represents the stretched exponential type at $t\ll\tau$ and interpolates between a normal relaxation, stretched exponential pattern, and terminal inverse power-law decay. The waiting time distribution of the continuous-time random walk can be attributed to energetic disorder. The energy in the space of appropriate phase–space coordinates exhibits numerous valleys that are consistent with the local energy minima (traps) and the particle must wait at the traps for the next jump (Fig. S1)^[27]^. On the other hand, the simple exponential relaxation assumes identical SD particles and involves a single, smooth energy barrier.

**Figure S1. Schematic illustration of the energy landscape (modified after Binder and Young^27^).** Black circles represent particles and arrows indicate particle jumps to the next site. Although the simple exponential relaxation involves only a single energy barrier, the stretched exponential function can be understood using models that involve an organized set of free-energy barriers.

**Fig. S2. Low-temperature measurements of tsunami boulders.** (A) FC and ZFC cycling curves for TB4. (B) Measurement of TB6 under the same conditions as those used in (A). (C) Cooling and warming of RT-IRM for TB4. (D) Measurement of TB6 under the same conditions as those used in (C).


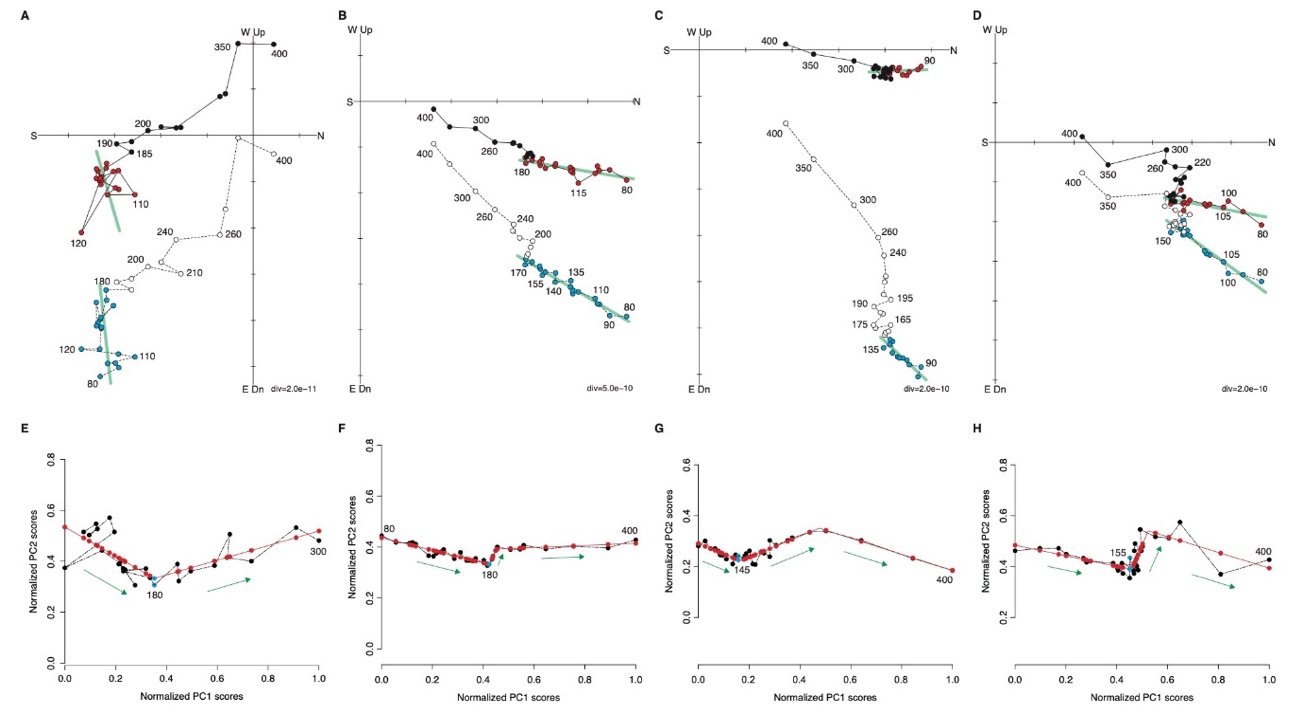


**Fig. S3. Vector plots and directional analysis of tsunami boulders.** (A–D) Vector plots of tsunami boulders: (A) TB2-1, (B) TB4-1, (C) TB4-3, (D) TB4-4. The black and red circles represent the horizontal direction, while white and blue circles represent the vertical direction. The red and blue circles represent the VRM component and green lines indicate the VRM direction obtained from PCA analysis. (E–H) Plots of PC1 scores and PC2 scores (black dots with lines) and segmented linear regression results (red dots with lines) for the datasets with scores normalized by the maximum and minimum scores of PC1 and PC2: (E) TB2-1, (F) TB4-1, (G) TB4-3, (H) TB4-4. Blue dots represent the inflection points.

**Fig. S4. Stereographic projection of the VRM direction.** VRM direction data were obtained from Fig. S1.

**Fig. S5. Boulder surface temperature variation.** The measurement was started at 08:45 (August 10, 2021; summer season) and ended at 18:00 (August 11, 2021). Air temperature data were obtained from the Japan Meteorological Agency.


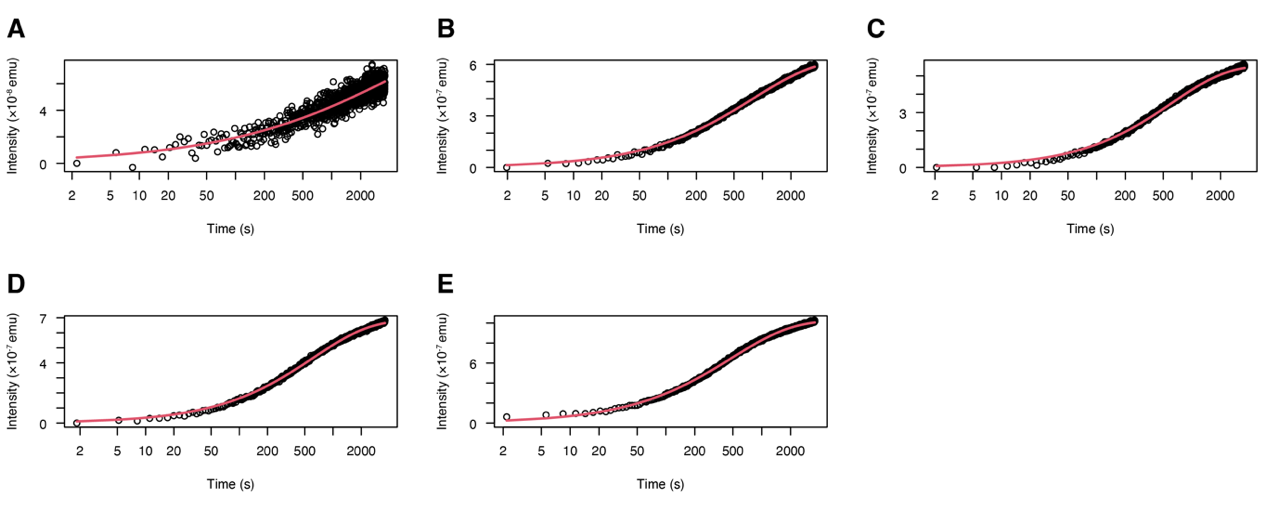


**Fig. S6. Time dependence of VRM.** White dots are data points and red lines are curve fitting results of the stretched exponential function. (A) TB2-1. (B) TB4-1. (C) TB4-1. (D) TB4-3. (E) TB4-4.

**Table S1. Summary of ^14^C dating results.**

| Sample ID | Part of the block | Sample code | 2σ | Median |
| --- | --- | --- | --- | --- |
| TB1 | Top | 20210527-1TOP | AD1645–1953 | AD1799 |
| TB1 | Bottom | 20210527-1BOTTOM | AD1568–1953 | AD1761 |
| TB2 | Top | 20210528-1TOP | AD629–1015 | AD822 |
| TB2 | Bottom | 20210528-1BOTTOM | AD602–990 | AD796 |
| TB3 | Top | 20210528-2TOP | AD1636–1953 | AD1795 |
| TB3 | Bottom | 20210528-2BOTTOM | AD1638–1953 | AD1796 |
| TB4 | Top | 20210528-3TOP | AD1577–1953 | AD1765 |
| TB4 | Bottom | 20210528-3BOTTOM | AD1580–1953 | AD1767 |
| TB5 | Top | 20210529-1TOP | AD1538–1943 | AD1741 |
| TB5 | Bottom | 20210529-1BOTTOM | AD1504–1918 | AD1711 |
| TB6 | Top | 20210529-2TOP | AD1597–1953 | AD1775 |
| TB6 | Bottom | 20210529-2BOTTOM | AD1584–1953 | AD1769 |
| TB7 | Top | 20210624-1SHIRAHO | AD1490–1900 | AD1695 |
| TB8 | Top | 20210624-2-1TOP | AD1644–1953 | AD1799 |
| TB8 | Bottom | 20210624-2-1BOTTOM | AD1602–1953 | AD1778 |
| TB8 | Top | 20210624-2-2TOP | AD1601–1953 | AD1777 |
| TB8 | Bottom | 20210624-2-2BOTTOM | AD1622–1953 | AD1788 |

**Supplementary References**

1. Néel, L. Theorie du trainage magnetique des ferromagnetiques au grains fins avec applications aux terres cuites. *Ann. Géophys.* **5**, 99-136 (1949).
2. Néel, L. Some theoretical aspects of rock magnetism. *Adv. Phys.* **4(14)**, 191-243 (1955).
3. Dunlop, D. J. Viscous magnetization of 0.04–100 μm magnetites. *Geophys. J. In.* **74(3)**, (1983).
4. Chamberlin, R. V., Mozurkewich, G. & Orbach, R. Time decay of the remanent magnetization in Spin-Glasses. *Phys. Rev. Lett*, **52**, 867-870 (1984).
5. Yu, Y. & Tauxe, L. Acquisition of viscous remanent magnetization. *Phys. Earth Planet. Inter.* **159(1-2)**, 32-42 (2006).
6. Dunlop, D. J. & Özdemir, Ö. Effect of grain size and domain state on thermal demagnetization tails. *Geophys. Res. Lett.* **27**, 1311-1314 (2000).
7. Street, R. & Woolley, J. C. A study of magnetic viscosity. *Proc. Physi. Soc.* **A62(9)**, 562-572 (1949).
8. Saito, R. & Murayama, K. A universal distribution function of relaxation in amorphous materials. *Sol. Stat. Commun.* **63**, 625-627 (1987).
9. Chamberlin, R. V. & Scheinfein, M. R. Slow relaxation in iron: A ferromagnetic liquid. *Science,* **260(5111)**, 1098-1101 (1993).
10. Williams, W. & Muxworthy, A. R. Understanding viscous magnetization of multidomain magnetite. *J. Geophys. Res.* **111(B2)**, B02102 (2006).
11. Montroll, E. W. & Bendler, J. T. On Lévy (or stable) distributions and the Williams-Watts model of dielectric relaxation. *J. Stat. Phys.* **34**, 129-162 (1984).
12. Lindsey, C. P. & Patterson, G. D. Detailed comparison of the Williams–Watts and Cole–Davidson functions. *J. Chem. Phys.* **73**, 3348 (1980).
13. Mainardi, F. *Fractional calculus and waves in linear viscoelasticity: An introduction to mathematical models* (Imperial College Press, London, 2010).
14. Humbert, P. Nouvelles correspondences symboliques. *Bull. Soc. Math. Fr.* **69**, 121-129 (1945).
15. Pollard, H. The representation of $e^{-x^{\lambda}}$ as a Laplace integral. *Bull. Amer. Math. Soc.* **52**, 908–910 (1946).
16. Sokolov, I. M. & Klafter, J. Field-induced dispersion in subdiffusion. *Phys. Rev. Let.* **97**, 140602 (2006).
17. Langlands, T. A. M. & Henry, B. I. Fractional chemotaxis diffusion equations. *Phys. Rev. Lett.* *E.* **81**, 051102 (2010).
18. Montroll, E. W. & Weiss, G. H. Random walks on lattices. II. *J. Math. Phys.* **6**, 167 (1965).
19. Reeves, D. B. & Weaver, J. B. Approaches for modeling magnetic nanoparticle dynamics. *J. Crit. Rev. Biomed. Eng.* **42(1)**, 85-93 (2014).
20. Scher, H. & Montroll, E. W. Anomalous transit-time dispersion in amorphous solids. *Phys. Rev. B.* **15**, 2455 (1975).
21. Condamin, S., Bénichou, O. & Klafter, J. First-passage time distributions for subdiffusion in confined geometry. *Phys. Rev. Lett.* **98**, 250602 (2007).
22. Sokolov, I. G., Klafter, J. & Blumen, A. Fractional kinetics. *Phys. Today.* **55**, 48 (2002).
23. Metzler, R., Barkai, E. & Klafter, J. Anomalous diffusion and relaxation close to thermal equilibrium: A fractional Fokker-Planck equation approach. *Phys. Rev. Lett.* **82**, 3563 (1999).
24. Metzler, R. & Klafter, J. The restaurant at the end of the random walk: Recent developments in the description of anomalous transport by fractional dynamics. *J. Phys. A: Math. Gen.* **37**, R161 (2004).
25. Metzler, R. & Klafter, J. The random walk guide to anomalous diffusion: A fractional dynamics approach. *Phys. Rep.* **339**, 1-77 (2000).
26. Metzler, R. & Klafter, J. From stretched exponential to inverse power-law: Fractional dynamics, Cole–Cole relaxation processes, and beyond. *J. Non-Cryst. Sol.* **305**, 81-87 (2002).
27. Binder, K. & Young, A.P. Spin glasses: Experimental facts, theoretical concepts, and open questions. *Rev. Mod. Phys.* **58**, 801 (1986).
